# The HIV proteins Tat and Nef promote human bone marrow mesenchymal stem cell senescence and alter osteoblastic differentiation

**DOI:** 10.1111/acel.12308

**Published:** 2015-04-07

**Authors:** Carine Beaupere, Marie Garcia, Jerome Larghero, Bruno Fève, Jacqueline Capeau, Claire Lagathu

**Affiliations:** 1 Sorbonne Universités, UPMC Univ Paris 06, UMR_S 938CDR Saint-Antoine, F-75012, Paris, France; 2 INSERM, UMR_S 938CDR Saint-Antoine, F-75012, Paris, France; 3 Institute of Cardiometabolism and NutritionParis, France; 4 Inserm, UMR1160, Institut Universitaire d’Hématologie, Hôpital Saint-Louis75010, Paris, France; 5 AP-HP, Unité de Thérapie Cellulaire et CIC de Biothérapies, Hôpital Saint LouisParis, France; 6 Univ Paris DiderotSorbonne Paris Cité, F-75475, Paris, France; 7 APHP, Hôpital Saint-AntoineF-75012, Paris, France; 8 APHP, Hôpital TenonF-75020, Paris, France

**Keywords:** HIV proteins, senescence, oxidative stress, inflammation, autophagy, osteoblastic differentiation

## Abstract

To maintain bone mass turnover and bone mineral density (BMD), bone marrow (BM) mesenchymal stem cells (MSCs) are constantly recruited and subsequently differentiated into osteoblasts. HIV-infected patients present lower BMD than non-HIV infected individuals and a higher prevalence of osteopenia/osteoporosis. In antiretroviral treatment (ART)-naive patients, encoded HIV proteins represent pathogenic candidates. They are released by infected cells within BM and can impact on neighbouring cells. In this study, we tested whether HIV proteins Tat and/or Nef could induce senescence of human BM-MSCs and reduce their capacity to differentiate into osteoblasts. When compared to nontreated cells, MSCs chronically treated with Tat and/or Nef up to 30 days reduced their proliferative activity and underwent early senescence, associated with increased oxidative stress and mitochondrial dysfunction. The antioxidant molecule N-acetyl- cysteine had no or minimal effects on Tat- or Nef-induced senescence. Tat but not Nef induced an early increase in NF-κB activity and cytokine/chemokine secretion. Tat-induced effects were prevented by the NF-κB inhibitor parthenolide, indicating that Tat triggered senescence via NF-κB activation leading to oxidative stress. Otherwise, Nef- but not Tat-treated cells displayed early inhibition of autophagy. Rapamycin, an autophagy inducer, reversed Nef-induced senescence and oxidative stress. Moreover, Tat+Nef had cumulative effects. Finally, Tat and/or Nef decreased the MSC potential of osteoblastic differentiation. In conclusion, our *in vitro* data show that Tat and Nef could reduce the number of available precursors by inducing MSC senescence, through either enhanced inflammation or reduced autophagy. These results offer new insights into the pathophysiological mechanisms of decreased BMD in HIV-infected patients.

## Introduction

Bone remodelling is controlled by the balance between osteoblast-mediated bone deposition and osteoclast-mediated bone resorption. Under pathophysiological conditions, this balance is affected and leads to abnormal bone mass turnover and to bone loss. Mesenchymal stem cells (MSCs) are multipotent precursors, with the capacity to differentiate towards multiple tissue lineages such as adipocytes, chondroblasts and osteoblasts (Verma *et al*., 2002; Kim *et al*., 2012). To achieve osteogenic differentiation and maintain bone mass, MSCs within the bone marrow (BM) proceed through a number of functional stages including proliferation, matrix maturation and mineralization. Osteoporosis and osteopenia are bone disorders characterized by a loss of bone mass and consequent reduction in bone strength, which in turn leads to an increased risk of fractures (Mazziotti *et al*., 2010). While a certain degree of bone weakening with age is considered normal, several clinical studies have shown that HIV-infected patients present an increased prevalence of osteopenia ranging from 20% to 50% and of osteoporosis reaching up to 20% (Powderly, 2002; Brown & Qaqish, 2006; Stone *et al*., 2010). It is now clearly established that HIV infection is an independent risk factor for osteopenia and osteoporosis (Dolan *et al*., 2006; Stone *et al*., 2010). Cross-sectional studies of ART-naive HIV-infected patients found altered levels of bone turnover markers, in accordance with a reduced level of bone formation when compared to HIV-uninfected controls (Haskelberg *et al*., 2011).

Currently, HIV infection is controlled by efficient ART, transforming this situation into a chronic disease. However, elderly HIV-infected patients often present an increased incidence of age- related comorbidities, including significant reductions in bone mineral density (BMD). These observations led to propose that HIV-infected patients suffer from accentuated aging (Capeau, 2011). The increased risk of osteoporosis in HIV-infected patients probably results from a combined effect of ART and HIV infection, in addition to other classical risk factors as drug consumption, low body weight and immune dysfunction (Stone *et al*., 2010; Castronuovo *et al*., 2013). To better understand the pathophysiology of bone loss in these patients is an important challenge.

Clinical studies have established that several HIV-related factors, including HIV infection and chronic inflammation, affected the balance between bone formation and resorption (Aukrust *et al*., 1999; Haskelberg *et al*., 2011). As MSCs express the CD4 receptors and CCR5 and CXCR4 coreceptors, it is conceivable that these cells are susceptible to HIV infection, although integrated proviruses are rarely found and a productive infection has not yet been documented (Nazari-Shafti *et al*., 2011). Nonetheless, hematopoietic progenitor cells (HPCs) in the BM of HIV-infected individuals have been proposed as a persistent HIV reservoir (McNamara *et al*., 2013), releasing HIV proteins or infectious virions that could induce bystander harmful effect on surroundings cells, such as apoptosis, oxidative stress, mitochondrial dysfunctions or autophagy alterations (Kyei *et al*., 2009; Raymond *et al*., 2011; Debaisieux *et al*., 2012). Proximity of MSCs and infected HPCs in the BM niche makes MSCs a direct target of the HIV proteins. Some HIV proteins such as gp120, Gag, Tat and Rev have been shown to affect osteoblastic differentiation and activity *in vitro* (Cotter *et al*., 2007, 2011; Gibellini *et al*., 2010).

Aging MSCs display reduced proliferative and osteoblastic differentiation capacities (Fehrer & Lepperdinger, 2005; Zhou *et al*., 2008), which contribute to decreased bone formation. Therefore, we hypothesized that bystander effects of HIV proteins arising from neighbouring infected cells or from reservoirs may abrogate marrow MSC self-renewal by inducing early aging and affecting differentiation properties. We chose to study two HIV proteins secreted by infected cells in the extracellular medium: Tat (trans-activator of transcription), which is essential for transcriptional activation and able to recruit and activate NF-κB (nuclear factor κ-light-chain-enhancer of activated B cells) (Fiume *et al*., 2012; Ju *et al*., 2012), and Nef (negative-regulating factor), an accessory protein protecting HIV from degradation by acting as an anti-autophagic maturation factor (Dinkins *et al*., 2010; Raymond *et al*., 2011). We tested whether Tat and/or Nef could induce premature senescence of osteoblast precursor stem cells, namely MSCs, which in turn could be involved in osteoblast depletion and bone loss.

## Results

### Long term treatment with Tat and/or Nef inhibits proliferation and induces senescence of MSCs

**Fig 1:**
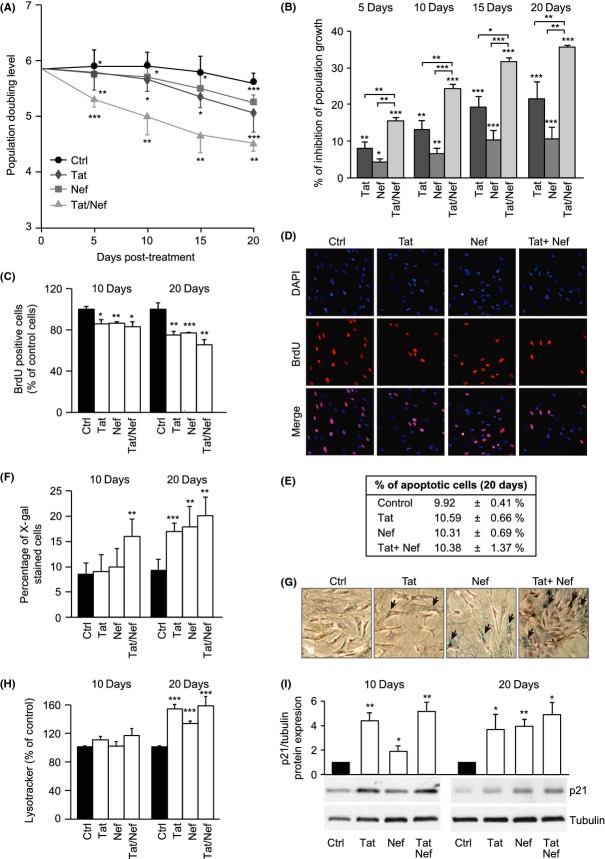
Long-term exposure to Tat and/or Nef inhibits proliferation and induces senescence in mesenchymal stem cells (MSCs). The population doubling level (PDL) was calculated as stated in Experimental procedures. Mean PDL values (± SEM) were determined at the indicated days post- HIV-protein treatment (A). The per cent inhibition of population growth was calculated for each HIV protein by determining the increase in total cell number that occurred after 20 days of continuous exposure to HIV proteins, compared to control cells (B). Cultures of nonconfluent MSCs on coverslips were fixed, stained with BrdU and analysed by immunofluorescence microscopy to determine the number of dividing cells after day 10 and day 20 of HIV-protein treatment (C). Representative micrographs of BrdU positive cells and DAPI-stained cells at day 20 are shown (D). The per cent of apoptotic cells was determined as stated in Experimental procedures (E). Senescence was evaluated by SA-β-galactosidase activity and expressed as the total per cent of SA-β-galactosidase positive cells at pH6 (F). Representative micrographs of SA-β-galactosidase positive cells are shown (G). Lysosomal accumulation was assessed by LysoTracker fluorescence probe and expressed as per cent of control cells (H). Whole-cell lysates, extracted from MSCs at day 10 and day 20 of HIV-proteins treatment, were analysed by immunoblotting. Representative immunoblots of cell cycle arrest markers p21^WAF1^ and tubulin (loading control) are shown (I). Results are mean ± SEM. All experiments were performed in duplicate or triplicate in MSCs isolated from 3 to 4 different bone marrow donors. *P < 0.05, **P < 0.01,***P < 0.001, vs control cells.

We first evaluated whether chronic exposure for 20 days to Tat and/or Nef affected the proliferation capacities of MSCs *in vitro*. Human MSCs treated with Tat and/or Nef displayed a reduced proliferative activity that worsened with increasing cellular passages (Fig.1A). Population doubling level (PDL) declined from day 5 to day 20 in the presence of the HIV proteins. After 20 days, Tat- treated MSC displayed a 21.4 ± 4.5% inhibition in population growth compared to controls, whereas Nef-treated cells displayed a 10.6 ± 3.0% inhibition (Fig.1B). Moreover, we observed an early cumulative effect of Tat+Nef with an inhibition of proliferation of 35.5 ± 0.4% after 20 days. Reduced proliferation was confirmed by a decreased number of proliferative cells labelled with BrdU at day 10 and day 20 (Fig.1C,D). Apoptosis was not increased, when assayed by caspase 3/7 activity (Fig.1E).

In control cells, the percentage of senescent cells X-gal stained was 8.6 ± 2.2% and 9.3 ± 2.2%, after 10 and 20 days of culture, respectively (Fig.1F,G). After 10 days, senescence was increased in cells treated with Tat+Nef, and after 20 days, its level was of 17.1 ± 1.6%, 18.0 ± 4.1% and 20.2 ± 3.7% in Tat-, Nef- and Tat+Nef-treated cells, respectively (Fig.1F). Accordingly, the HIV proteins increased lysosome accumulation, a hallmark of aging, after 20 but not 10 days (Fig.1H). Finally, we also found an increased protein expression of the cell cycle arrest marker p21^WAF1^ after 10 and 20 days of treatment (Fig.1I). Taken as a whole, these results show that Tat- or Nef- treated cells presented an increased onset on cell senescence after 20 days. Moreover, Tat+Nef induced an early onset of senescence.

To confirm the specificity of the effect of Tat and/or Nef, we performed experiments in the presence of mutated proteins Tat*Mut, mutated in the transactivation domain, and Nef*Mut, a signalling-defective mutant (Fig. S1). Tat*Mut displayed little effect on population growth (Fig. S1A,B), senescence (Fig. S1D) and Lysotracker (Fig. S1E), as compared to controls. Nef*Mut had no effect on cell proliferation (Fig. S1 A–C), cellular senescence (Fig.S1D) or Lysotracker (Fig.S1E) in contrast to nonmutated Nef.

We also treated the MSCs up to day 20 with the viral proteins and then removed, or not, these proteins to evaluate the potential reversion of the alterations (Fig. S2). As shown in Figures S2 A and B (Supporting information), the removal of Tat and/or Nef was associated with a rapid recovery of population growth, which results from a significant attenuation in senescent cell generation (Fig. S2C,D), at day 30. These results suggest the direct involvement of Tat and Nef in the observed alterations.

### Long-term treatment with Tat and/or Nef induces oxidative stress and mitochondrial dysfunction

**Fig 2:**
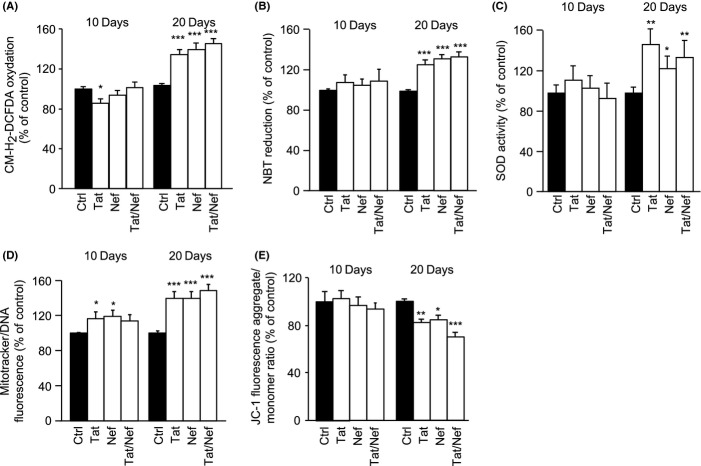
Tat and/or Nef induce oxidative stress and mitochondrial dysfunction in MSCs. MSCs were treated with the HIV proteins Tat and/or Nef up to 20 days. ROS production was assessed by the oxidation of CM-H_2_DCFDA (A) and the reduction of NBT (B) and expressed as % of control cells. Superoxide dismutase activity was evaluated as stated in Experimental procedures and expressed as per cent of control cells (C). Mitochondrial mass was evaluated by MitoTracker Red-Probe, and results are expressed as per cent of control cells (D). The cationic dye JC-1 evaluates mitochondrial membrane potential, monomers (green fluorescence) and aggregates (red–orange fluorescence) were quantified. The results are expressed as the ratio of aggregate/monomer fluorescence (E). Results are mean ± SEM. All experiments were performed in duplicate or triplicate. *P < 0.05, **P < 0.01,***P < 0.001, *vs* control cells.

The production of reactive oxygen species (ROS), measured by CM-H_2_-DCFDA oxidation and the reduction of nitroBlue tetrazolium (NBT), which measures the cellular oxidase activity, was unchanged after 10 days of treatment with the HIV proteins, but increased after 20 days by 1.3- to 1.4-fold by Tat and/or Nef, when compared to control cells (Fig.2A,B) along with an increased superoxide dismutase activity (SOD) (Fig.2C). To search for mitochondrial dysfunction, we evaluated mitochondrial volume by using Mitotracker Red labelling of mitochondria. We observed that the volume of mitochondria on day 10 was mildly increased in Tat and/or Nef-treated MSCs and reached up to 1.5-fold of control on day 20 (Fig.2D). We observed a destabilization of the mitochondrial membrane potential, as shown by the JC1 test (Fig.2E), with a 20% decrease in JC-1 fluorescence in Tat- or Nef-treated cells, and a 30% decrease in Tat+Nef treated cells after 20 days, in accordance with the onset of mitochondrial dysfunction.

Tat*Mut and Nef*Mut displayed no effect on oxidative stress or mitochondrial dysfunctions (Fig. S1F–H) at both day 10 and day 20. The removal of the HIV proteins at day 20 was associated with the normalization of oxidative stress and mitochondrial dysfunctions at day 30 (Fig. S2E–G). Thus, these complementary experiments confirm that Tat and Nef have a direct impact on oxidative stress and mitochondrial dysfunctions in MSCs.

### An antioxidant treatment prevents Tat- and/or Nef-induced oxidative stress

**Fig 3:**
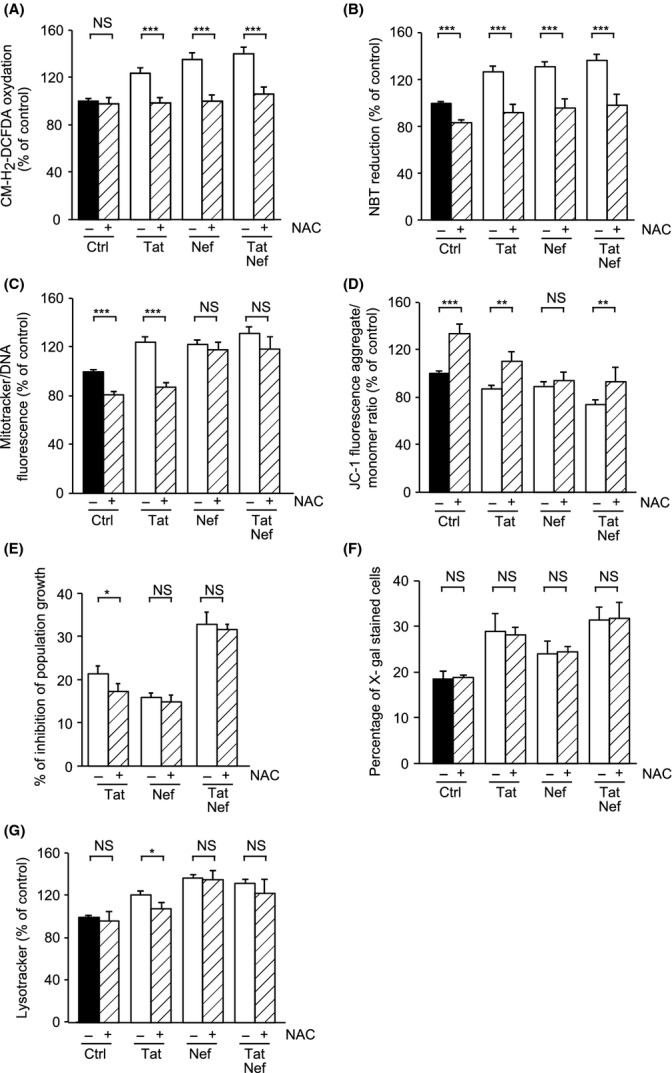
NAC treatment prevents oxidative stress and partially prevents Tat-induced senescence in MSCs. NAC was added, or not, at day 10, for 10 days. ROS production was assessed on day 20 post-HIV-protein treatment by the oxidation of CM-H_2_DCFDA (A) and the reduction of NBT (B). Mitochondrial mass was evaluated by MitoTracker Red-Probe (C), and mitochondrial membrane potential by JC-1 fluorescent dye (D). Mean cell number and the per centinhibition of population growth were calculated for each condition (E). Senescence was evaluated by SA-β-galactosidase activity and expressed as the total per cent of SA-β-galactosidase cells at pH6 (F). Lysosomal accumulation was assessed by LysoTracker fluorescence probe and expressed as Per cent of control cells (G). Results are mean ± SEM. All experiments were performed in duplicate or triplicate. *P < 0.05, **P < 0.01,***P < 0.001, *vs* control or *vs* Tat- and/or Nef-treated cells without NAC. NS, nonsignificant.

To assess whether ROS production was involved in the onset of cellular senescence, MSCs were treated from day 10 to day 20 with the antioxidant N-acetyl cysteine (NAC). As shown in Fig.3A,B, on day 20, NAC prevented increased ROS production induced by Tat and/or Nef. NAC also normalized altered mitochondrial volume and function induced by Tat but not by Nef (Fig.3C,D). Regarding senescence, NAC mildly prevented Tat-decreased proliferation and lysosome accumulation, but did not change SA-β-galactosidase activity (Fig.3E–G). This suggests that even if oxidative stress participates to Tat-related dysfunction, its role in the induction of Tat-related senescence is mild if any. In parallel, NAC did not prevent Nef-induced decrease in cell proliferation, lysosome accumulation and SA-β-galactosidase activity (Fig.3E–G). Overall, these results show that oxidative stress participated to Tat- but not to Nef-related mitochondrial dysfunction and not, or mildly, to Tat- or Nef-induced senescence.

### Tat but not Nef induces inflammation: reversion of Tat effects by the NF-κB inhibitor parthenolide

**Fig 4:**
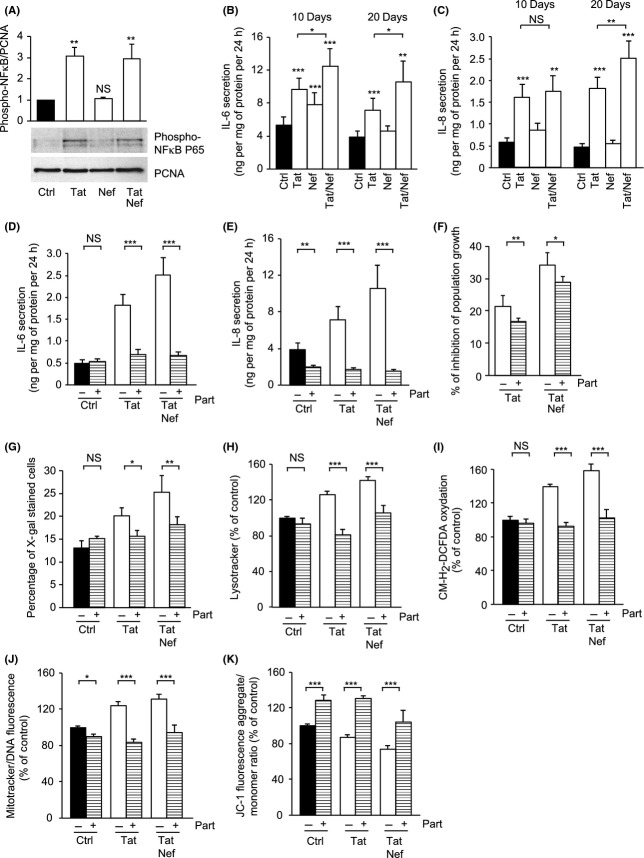
Tat but not Nef induces a pro-inflammatory profile: reversion by the NF-κB inhibitor parthenolide. Representative immunoblots of nuclear phospho-p65-NF-κB (ser536) and PCNA/proliferating cell nuclear antigen (loading control) at day 20 (A) are shown. Upon day 10 and day 20, the levels of IL-6 (B) and IL-8 (C) in the cell culture media were determined with ELISA assays. Parthenolide (Part) was added, or not, at day 18 for 48 h. IL-6 (D) and IL-8 (E) in the cell culture media were determined. Results are expressed as ng/mg of total cell proteins 24 h. Mean cell number and the per cent inhibition of population growth were calculated for each condition (F). Senescence was evaluated by SA-β-galactosidase activity and expressed as the total per cent of SA-β-galactosidase cells at pH6 (G). Lysosomal accumulation was assessed by LysoTracker fluorescence probe and expressed as per cent of control cells (H). ROS production was assessed on day 20 by the oxidation of CM-H_2_DCFDA (I). Mitochondrial mass was evaluated by MitoTracker Red-Probe (J), and mitochondrial membrane potential by JC-1 fluorescent dye (K). Results are mean ± SEM. All experiments were performed in duplicate or triplicate. *P < 0.05, **P < 0.01,***P < 0.001, *vs* control or *vs* Tat- and/or Nef-treated cells without parthenolide. NS, nonsignificant.

Treatment with Tat but not Nef resulted in an increased nuclear translocation of the activated pro- inflammatory and pro-senescent transcription factor NF-κB, as shown by the accumulation of the activated phospho-Ser 536 form of p-65 in the nucleus (Fig.4A). In agreement, Tat- but not Nef- treated MSCs displayed an inflammatory phenotype, as shown by the increased secretion of IL-6 and IL-8 (Fig.4B,C) observed after 10 days and persistent at 20 days.

To evaluate whether in Tat-treated cells, the early activation of NF-κB and inflammatory markers could be responsible for the onset of cellular senescence, we used parthenolide, an NF-κB inhibitor, over a short period of time (48 h) on MSCs treated for 18 days with Tat or Tat+Nef. As expected, treatment with the NF-κB inhibitor was able to prevent the increased secretion of IL-6 and IL-8 (Fig.4D,E) induced by Tat and Tat+Nef. Regarding senescence, parthenolide significantly reduced the inhibition of cell population proliferation induced by Tat (from 21.4 ± 3.2% to 16.7 ± 1.1%) and Tat+Nef (from 34.2 ± 3.9% to 28.9 ± 1.6%) (Fig.4F) and normalized the percentage of senescent cells in Tat- and Tat+Nef-treated cells to about 15% (Fig.4G) and lysosome accumulation (Fig.4H). Moreover, parthenolide prevented ROS production (Fig.4I) and mitochondrial dysfunction in Tat- and Tat+Nef-treated MSCs (Fig.4J,K). Taken as a whole, these results showed that Tat exerted its effect via the activation of NF-κB, which induced the secretion of cytokines/chemokines leading to cellular senescence and oxidative stress.

### Nef but not Tat decreases autophagy: prevention of Nef-induced effects by rapamycin

**Fig 5:**
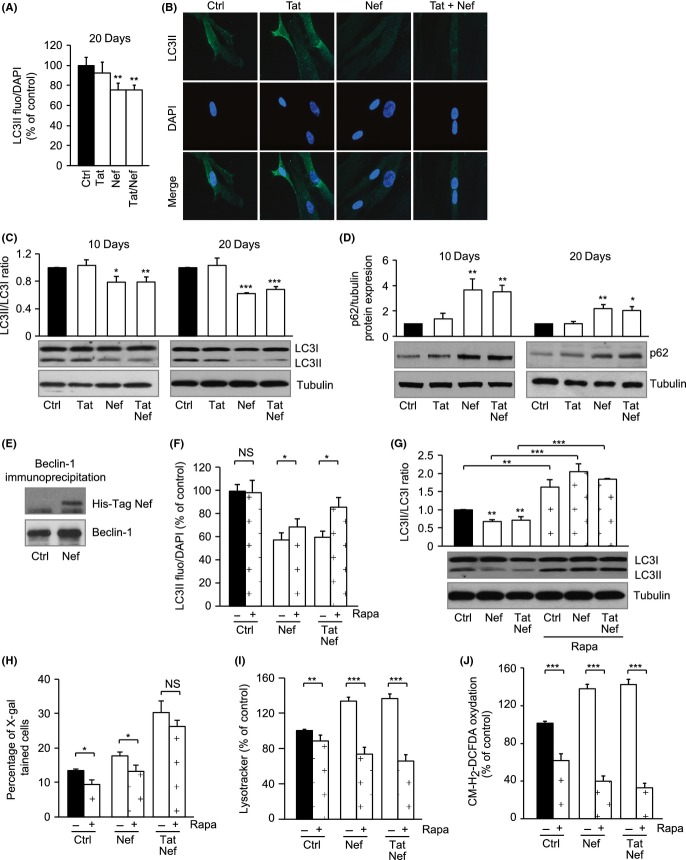
Nef impedes the autophagic efflux in MSCs: prevention by rapamycin. MSCs were treated with the HIV proteins Tat and Nef for up to 20 days. The level of autophagic activity was determined by LC3-II staining of autophagic vesicles, as stated in Experimental procedures. Total fluorescence of LC3-II was evaluated, and results are expressed as per cent of control cells (40× magnification) (A). Representative micrographs of LC3-II-labelled and DAPI-stained cells are shown (100× magnification) (B). Whole-cell lysates were extracted from MSCs at day 10 and day 20 of treatment with the HIV proteins and analysed by immunoblotting. Representative immunoblots of LC3 and tubulin (loading control) and the relative quantification of LC3II/I ratio are shown (C). Representative immunoblots of p62 and tubulin (loading control) are shown (D). Whole- cell lysates were extracted from MSCs at day 20 of treatment with histidine-tagged Nef, and Beclin-1 was immunoprecipitated. Representative immunoblots of Nef (using an anti-his-tag antibody) and beclin-1 are shown (E). Rapamycin (Rapa) was added from day 15 to day 20. The level of autophagic activity was determined by with LC3-II staining of autophagic vesicles, as stated in Experimental procedures. Total fluorescence of LC3-II was evaluated and results are expressed as % of control cells (F). Representative immunoblots of LC3 and tubulin (loading control) and the relative quantification of LC3II/I ratio are shown (G). Senescence was evaluated by SA-β- galactosidase activity and expressed as the total percentage of SA-β-galactosidase cells at pH6 (H). Lysosomal accumulation was assessed by LysoTracker fluorescence probe and expressed as percentage of control cells (I). ROS production was assessed on day 20 by the oxidation of CM-H_2_DCFDA (J). Results are mean ± SEM. All experiments were performed in duplicate or triplicate. *P < 0.05, **P < 0.01,***P < 0.001, *vs* control or *vs* Tat- and/or Nef-treated cells without rapamycin. NS, nonsignificant.

After 20 days of treatment, Nef- but not Tat-treated cells showed a decreased LC3II labelling of the autophagosomes of about 25% as compared to the control (Fig.5A,B). In accordance, we observed in Nef- but not in Tat-treated cells a decreased conversion of LC3I to LC3II reflecting a decrease in autophagic efflux from day 10 (Fig.5C), and the accumulation of the p62 protein (Fig.5D), a marker of autophagy inhibition. In addition, Nef decreased the mRNA expression of autophagy-inducing proteins such as ULK1, BECN1 and GABARAP (Fig. S3A). Moreover, we observed a direct interaction between Nef (His-tagged) and Beclin-1, which were co- immunoprecipitated (Fig.5E), suggesting that Nef could inhibit autophagy through a direct interaction with Beclin-1.

We also highlighted that Nef-induced decrease in autophagy occurred together with a hyperactivation on day 10 and day 20 of protein kinase B (Akt/PKB) (Fig. S3B), a kinase acting upstream of mTOR, implicated in the inhibition of autophagy in response to IGF1 (Nair & Ren, 2012).

To evaluate whether Nef-induced inhibition of autophagy was a primary event leading to senescence, we used rapamycin, an immunosuppressant that acts through the inhibition of mTOR, thus activating autophagy (Markaki & Tavernarakis, 2013). When rapamycin was added to MSCs from day 15 to day 20 of treatment with Nef and Tat+Nef, we observed a decrease in MSC population growth, as expected owing to the known antiproliferative activity of rapamycin (Fig. S3C). Despite this effect, rapamycin treatment increased the amount of the autophagy marker LC3II (Fig.5F) and restored the LC3I to LC3II conversion (Fig.5G). Rapamycin decreased the level of senescence as shown by the reduced percentage of senescent cells in control and Nef-treated MSCs by 25% (Fig.5H) and the reduced volume of lysosomes by about 45% for Nef and 52% for Tat+Nef (Fig.5I). Rapamycin also decreased the ROS production induced by Nef and Tat+Nef by 74% and 77% (Fig.5J) and mitochondrial content by 33% and 49% (Fig. S3D), and increased the mitochondrial membrane potential (Fig. S3E). These results showed that Nef inhibited autophagy, possibly in part through a direct interaction with Beclin-1, leading to the onset of cellular senescence and oxidative stress.

### Long-term treatment with Tat and/or Nef alters MSC osteoblastic differentiation

**Fig 6:**
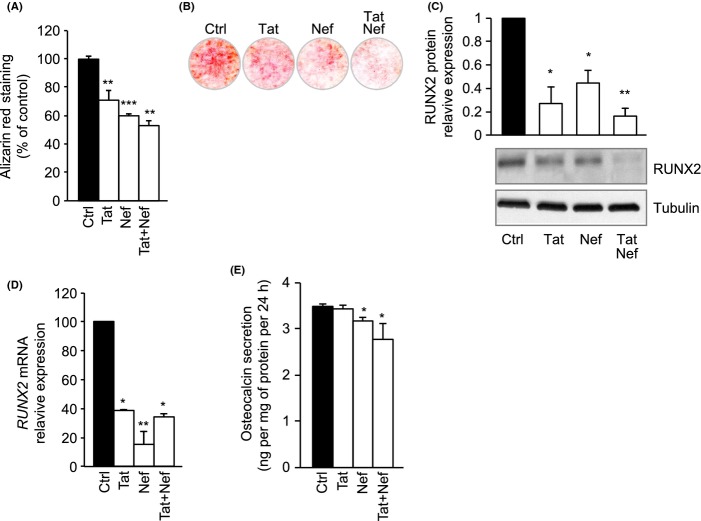
Tat- and Nef-induced senescence is associated with altered osteoblastic potential of MSCs. MSCs were differentiated into osteoblasts after 20 days of treatment with Tat and/or Nef. To evaluate the osteoblastic potential of MSCs, cells were stained with Alizarin Red 15 days post induction of differentiation. Quantification of Alizarin Red is expressed as per cent of control cells (A), and representative micrographs are shown (B). Whole-cell lysates were extracted at day 15 post induction of differentiation from MSCs and analysed by immunoblotting. Representative immunoblots of RUNX2 and ERK1/2 are shown (C). Human *RUNX2 and OSX* mRNA levels were measured using real time RT–PCR. The relative mRNA expression levels were normalized to PPIA (D). Osteocalcin in the cell culture media was determined with ELISA assays. Results are expressed as pg/mg of total cell proteins-24 h (E). Results are mean ± SEM. All experiments were performed in duplicate or triplicate. *P < 0.05, **P < 0.01,***P < 0.001, *vs* control cells.

Age-related loss of bone mass is associated with altered differentiation capacities affecting osteoblastic fate. Thus, we undertook a study to evaluate the impact of Tat and/or Nef on the ability of MSCs to differentiate into mature osteoblasts. After a 20-day pretreatment with Tat and/or Nef, confluent cells were induced to differentiate in a pro-osteoblastic media, in the presence of the HIV proteins. After 15 days of differentiation, a decreased staining with alizarin red, which visualizes calcium deposition, was observed in MSCs long-term treated with Tat and/or Nef (Fig.6A,B). Accordingly, the decreased ability of MSCs to undergo osteoblastic differentiation in the presence of the HIV proteins was supported by the lower level of the RUNX2 protein (Fig.6C), decreased RUNX2 mRNA expression (Fig.6D) and also a decreased level of osteocalcin secretion (Fig.6E).

## Discussion

We show here that the HIV proteins Tat and Nef can induce cellular senescence and oxidative stress in BM-MSCs, leading to a reduced capacity of differentiation towards the osteoblastic lineage. Importantly, for the first time, we demonstrate that Tat-induced senescence was mediated by the activation of the NF-κB pathway, while Nef-induced senescence resulted from the inhibition of autophagy. We propose that Tat and Nef, at low concentrations, could alter the osteoblastic precursors, namely MSCs, giving new insights into the decreased BMD observed in HIV-infected patients.

We evaluated here the impact of the HIV proteins Tat and Nef, as both are known to be secreted by infected cells into the extracellular media and suspected to induce bystander cellular damage to the noninfected surroundings cells (Kyei *et al*., 2009; Raymond *et al*., 2011; Debaisieux *et al*., 2012). Indeed, within the BM, MSCs could be the indirect target of HIV infection.

We observed, at first, that both Tat and Nef induced cellular senescence. Moreover, Tat and Nef cotreatment had a cumulative effect related to the different signalling pathways affected by Tat and Nef. Decreased population growth was a sensitive marker as it can be observed as soon as after 5 days of treatment, while increased senescence occurred only after 10 days when Tat and Nef were added together, or 20 days for SA-β-galactosidase activity induced by Tat or Nef alone and for lysosome accumulation.

Many studies have shown that oxidative stress plays a major role in MSC alterations (Stolzing *et al*., 2008; Kasper *et al*., 2009; Brandl *et al*., 2011). We observed that increased ROS production occurred latter that the decreased cell proliferation, in response to the HIV proteins. The anti oxidant molecule NAC reduced ROS production in both Tat- and Nef-treated MSCs as expected; however, it could not prevent any of the other effects induced by Nef alone. Therefore, we propose that Nef-induced oxidative stress is a late event induced by senescence or is induced independently of senescence. Otherwise, NAC prevented the effect of Tat on mitochondrial dysfunction, but had a mild effect, if any, on population growth and the level of lysosomes, indicating that the ability of Tat to induce senescence is mainly not dependant upon oxidative stress.

When evaluating the mechanisms whereby Tat altered MSCs, we observed that cytokine secretion and NF-κB activation were affected after 10 days, in accordance with the literature, indicating that Tat mediates part of its effect through the activation of NF-κB (Mahieux *et al*., 2001; Zhang *et al*., 2011). A common feature of aging tissues is low-level chronic inflammation, termed inflammaging. The transcription factor NF-κB has been shown to play a role in the induction of senescence (Tilstra *et al*., 2011). In accordance, senescent cells secrete proinflammatory cytokines and chemokines (such as IL-8 and IL-6) representative of a senescence-associated secretory phenotype (Coppe *et al*., 2008). NF-κB has been implicated in apoptosis, cell cycle progression, cell senescence and inflammation (Tilstra *et al*., 2011; Tchkonia *et al*., 2013). To assess its implication, we used parthenolide, a selective inhibitor of the IκB kinase, resulting in stabilization of the cytoplasmic IκBα, which in turn leads to inhibition of NF-κB nuclear translocation (Saadane *et al*., 2007). We found that all the effects of Tat were prevented by parthenolide, which allowed us to establish that NF-κB activation is a primary event in Tat-induced premature senescence.

Regarding Nef, previous studies have shown that Nef can inhibit autophagy through a direct interaction with Beclin-1, a protein required for the nucleation of the autophagosome (Kyei *et al*., 2009; Killian, 2012). Autophagy is the central degradation process in cells required for the recycling of damaged organelles and macromolecules (Rubinsztein *et al*., 2011). We confirmed here that Nef, but not Tat, could inhibit autophagy by using several markers including LC3I to LC3II conversion, mRNA expression of specific markers of autophagy initiation (ULK1) and autophagosome formation (Beclin1, GABARAP), accumulation of the protein p62 and an autophagic adaptor and reveal a direct interaction between Nef and Beclin-1. We also show that Nef could induce the activation of Akt, which activates mTOR leading to autophagy inhibition. Rapamycin, an activator of autophagy (Markaki & Tavernarakis, 2013), was able to prevent the effect of Nef on both oxidative stress and cellular senescence. Moreover, as Nef inhibits autophagy, it can also affect mitophagy, inducing dysfunctional mitochondria accumulation (Dinkins *et al*., 2010). This was also prevented in our study by the addition of rapamycin. Altogether, these results suggested that inhibition of autophagy could be the primary event in Nef-induced senescence.

Senescent MSCs are present in human BM of aged patients, presenting increased oxidative stress and decreased cell plasticity, affecting more particularly the osteoblast differentiation (Zhou *et al*., 2008). Thus, we evaluated the impact of Tat and/or Nef on the ability of MSCs to differentiate into mature osteoblasts. Early senescence, induced by Tat and Nef, was associated with a loss of the ability of MSCs to differentiate into osteoblasts, as shown by the decrease in several osteoblastic markers and calcium deposition. These results are in keeping with clinical data, showing a decreased BMD in HIV-infected patients (Powderly, 2002; Brown & Qaqish, 2006; Stone *et al*., 2010), stressing for a direct role of HIV in decreased bone formation.

Our study has limitations. It may not account for many factors involved in the accelerated pro- osteoporotic process observed in HIV-infected patients, meaning the HIV infection itself, immune balance, environmental factors and genetic predispositions, together with ART. As cellular senescence is a common pathway of many cell lineages other than MSCs, it is possible that Tat and Nef could induce senescence of several other cell types, as hematopoietic stem cells. The impact of Tat and Nef on osteoclasts, derived from the hematopoietic lineage, remains to be evaluated, as several studies indicate an increased level of resorption markers in HIV-infected patients (Barkhordarian *et al*., 2011; Haskelberg *et al*., 2011), emphasizing the role of HIV infection on bone destruction. Our data suggest that some HIV proteins could induce premature senescence of MSCs, therefore precluding the regeneration process normally associated with bone loss. However, we have not tested other HIV-1 proteins, which could also exert deleterious effects.

In conclusion, our data provide the first experimental support that HIV proteins could induce senescence of MSCs. It has been suggested that the regenerative capacity of tissues with high cell turnover, such as bone, might be reduced due to exhaustion of progenitor cells. We hypothesize that during HIV infection, induction of premature aging in MSCs, along with chronic inflammation and continuous oxidative stress, could contribute to cell attrition and MSCs exhaustion. Therefore, the consequence of early senescence induced by HIV would be the lack of an appropriate number of stem cells for further tissue replacement and the development of bone loss and aging. While Nef inhibited autophagy, we showed that Tat induced an inflammatory phenotype in MSCs, the two proteins exhibiting cumulative effects. Thus, our results support the idea that HIV-induced premature aging in MSCs, along with chronic inflammation, autophagy inhibition and sustained oxidative stress, could contribute to MSC exhaustion.

## Experimental procedures

### Cell culture and treatment

Experimental procedures, with human BM, have been approved by the Saint Louis Hospital Ethical Committees for human research (Paris, France), in accordance with the European Union guidelines and the Declaration of Helsinki. MSCs were isolated from washed filters used during BM graft processing. Healthy donors BM cells obtained after Ficoll separation (Invitrogen Corporation, San Diego, CA, USA) were cultured at the initial density of 5.10^4^ cells/cm^2^ in alpha-minimum essential medium, supplemented with 10% foetal bovine serum (Gibco, Invitrogen Corporation), 2 mmol/L glutamine, 2.5 ng/mL bFGF (PeproTech, Rocky Hill, NJ, USA) and Penicillin/Streptomycin (Gibco, Invitrogen Corporation) (Hernandez-Vallejo *et al*., 2013). Adherent cells were then trypsinized, harvested and cultured by seeding 5.10^3^ cells/cm^2^. Cultures were fed every 2 to 3 days and trypsinized every 5 days. BM-MSCs used in our study did not express hematopoietic antigens, such as CD34 and CD45, and were positive for CD73, CD44 and CD105 expression (Larghero *et al*., 2008; Freida *et al*., 2013). All experiments were performed on MSCs isolated from at least three different BM donors. Cells were exposed to HIV recombinant proteins Tat (Diatheva, Fano, Italy), His- tagged Nef (Jena Bioscience GmbH, Jena, Germany), mutated Tat Cys22 (Diatheva, Fano, Italy) or signalling-defective mutant Nef (Jena Bioscience GmbH, Jena, Germany) at the clinically relevant concentrations observed in HIV-infected patients naive of treatment of 40 ng/mL (Gougeon, 2003; Boya *et al*., 2004; Raymond *et al*., 2011), or to the solvent (PBS) for up to 30 days, from passage 3 to 8. Medium was changed every 2 to 3 days. In some experiments, the cells were also incubated for 10 days with 250 μmol/L N-acetyl-cysteine (NAC), or for 5 days with 100 nmol/L Rapamycin (Rapa) or for 2 days with 100 μmol/L parthenolide (Part) (all from Sigma-Aldrich, St Louis, MO, USA).

### Cell proliferation and apoptosis

Cellular senescence was evaluated by the PDL value calculated as log2 (D5/D0), where D0 and D5 are the number of cells at seeding and harvesting, respectively (Hernandez-Vallejo *et al*., 2013). Dividing cells were identified by measuring bromodeoxyuridine (BrdU) incorporation (BD Biosciences Pharmingen, San Diego, CA, USA). Upon day 9 and day 19 post-HIV-protein treatment, cells were incubated for 24 h with BrdU (15 μmol/L), then fixed and permeabilized. Anti-BrdU antibody (Santa Cruz Biotechnology, Santa Cruz, CA, USA) was revealed using secondary antibodies coupled to Texas Red (Jackson ImmunoResearch Laboratories, West Grove, PA, USA). Cell nuclei were visualized after diamidino-phenylindole hydrochloride staining (DAPI, Sigma-Aldrich). Dividing cells, examined by fluorescence microscopy, were counted in four randomly selected fields and expressed as a percentage of total cells. The percentage of apoptotic cells was determined using Apo-ONE homogeneous Caspase-3/7 assay (Promega Biosciences, San Luis Obispo, CA, USA). Quantification was performed on a plate fluorescence reader (Spectrafluor Plus, Tecan-France, Trappes, France) at 530 nm.

### Cellular senescence and autophagy

The positive blue staining of β-galactosidase was used as a biomarker of cellular senescence. Cells were incubated with appropriate buffer solution containing X-Gal (5-bromo-4-chloro-3-indolyl-β-D-galactopyranoside) (Sigma-Aldrich) (Hernandez-Vallejo *et al*., 2013). The blue-stained cells observed at pH 6 and pH 4 were counted in eight fields at 20× magnification, and the percentage of pH6 positive blue senescent cells was calculated. We used the acidotropic dye LysoTracker (Invitrogen Corporation) to evaluate lysosomal mass. Cells were cultured in 96-well plates, washed and incubated with Lysotracker (50 nmol/L) in DMEM, for 30 min at 37 °C in the dark. Quantification was performed on a plate fluorescence reader (Spectrafluor Plus) at 620 nm. Autophagosomes density was visualized by immunofluorescence with an antibody directed against LC3II (LC3, M152-3, MBL international, Woburn MA, USA) in MSCs cultured with the HIV proteins for 20 days (Leica TCS SP microscope, 40× and 100× magnifications). Nuclear DNA was stained with DAPI.

### Cytokine/chemokine and osteocalcin secretion

Human IL-6 and IL-8 concentrations in cell culture media were determined after 10 and 20 days of incubation with Tat and/or Nef, using Quantikine human sandwich ELISA kits (R&D Systems, Inc. Minneapolis, MN, USA). The sensitivity of the assays was: 0.7 pg/mL for IL-6 and 7.5 pg/mL for IL-8. Human osteocalcin concentration in cell culture media from differentiated MSCs was determined after 15 days of differentiation, by using Osteocalcin Human Direct ELISA Kit (Life technologies, Gaithersburg, MD, USA). The sensitivity of the assays was 0.4 ng/mL.

### Osteoblast differentiation

Differentiation into osteoblasts was triggered on MSCs in the presence of the HIV proteins, by culture in the osteoblast differentiation media for 15 days (10 mmol/L β-glycerophosphate, 50 μg/mL ascorbate). Cells were then stained for Alizarin Red (Sigma-Aldrich) (Hernandez-Vallejo *et al*., 2013).

### Mitochondrial dysfunctions and oxidative stress

The cationic dye JC-1 (tetrachloro-tetra-ethyl- benzimidazolyl-carbocyanine iodide) evaluated the mitochondrial membrane potential, and the MitoTracker Red probe (both from Invitrogen Corporation,) measured mitochondrial mass. Cells, cultured in 96-well plates, were incubated with JC-1 (4 μg/mL), or Mitotracker (50 nmol/L) in DMEM, for 60 min at 37 °C in the dark. Quantification was performed on a plate fluorescence reader (Spectrafluor Plus, Tecan-France, Trappes, France) at 595 and 530 nm (JC-1 aggregates and monomers, respectively) or 630 nm (MitoTracker). The production of ROS was assessed by the oxidation of 5-6-chloromethyl-2,7-dichlorodihydro-fluorescein diacetate (CM- H_2_DCFDA) (Invitrogen Corporation) or the reduction of nitroblue tetrazolium (NBT) (Sigma-Aldrich) (Hernandez-Vallejo *et al*., 2013). Results were normalized to cell protein content. Superoxide dismutase (SOD) activity was evaluated using a commercially available kit (Sigma-Aldrich).

### Protein extraction, immunoprecipitation and Western blotting

Whole-cell protein and nuclear protein were extracted and then electro-blotted on nitrocellulose membrane (Amersham Biosciences GE Healthcare Europe, Velizy Villacoublay, France). Specific proteins were detected by incubation with the appropriate primary antibodies (p21WAF1, Phospho p65 subunit of NF-κB (ser 536), p62, Akt, Phospho-Akt, RUNX2/CBFA, ERK1/2, PCNA) and horseradish-peroxidase- conjugated secondary antibodies. Immune complexes were detected by enhanced chemiluminescence (Amersham Biosciences GE Healthcare Europe, Velizy Villacoublay, France). Co-immunoprecipitation was performed on whole cellular extracts prepared in lysis buffer (Epitomics, Burlingame, CA, USA) using Beclin-1 specific antibody (Abcam, Cambridge, MA, USA) and Rabbit IgG-Dynabeads (Invitrogen Corporation). All washing steps were performed using a Dynamagnet, and the elution of Beclin-1 with its binding partners was performed by adding Laemmli buffer and boiling. Western blot was then performed using a His-tag specific antibody.

### RNA isolation and quantitative RT–PCR

Total RNA was isolated from cultured cells using an RNeasy kit (Qiagen, Valencia, CA, USA), and mRNA expression was analysed by RT–PCR (Hernandez-Vallejo *et al*., 2013). The sequence of the oligonucleotides used as primers is available upon request.

### Statistical analysis

All experiments were performed at least three times on triplicate samples. Data are expressed as means ± SEM. Statistical significance, between HIV-protein-treated cells vs control with or without parthenolide, rapamycin or NAC, was determined with nonparametric Wilcoxon’s test.

## Supporting Information

**Fig. S1** Complementary results: Effect of Mutated Tat and Nef. MSCs were treated with the nonmutated proteins Tat and/or Nef, Tat*Mut or Nef*Mut, up to 20 days. Mean PDL values (± SEM), were determined at the indicated days post-HIV-proteins treatment (A). The % inhibition of population growth was calculated for each HIV protein compared to control cells (B). BrdU incorporation was analysed by immunofluorescence microscopy to determine the number of dividing cells after a 20-day treatment with HIV-proteins mutated or not (C). SA-β-galactosidase activity was evaluated and expressed as the total % of positive cells at pH6 (D). Lysosomal accumulation was assessed by LysoTracker fluorescence probe and expressed as % of control cells (E). ROS production was assessed by the oxidation of CM-H_2_DCFDA (F) and expressed as % of control cells. Mitochondrial mass was evaluated by MitoTracker Red-Probe (G), and mitochondrial membrane potential by JC-1 fluorescent dye (H). Results are mean ± SEM. All experiments were performed in duplicate or triplicate in MSCs isolated from 3 different bone marrow donors. **P* < 0.05, ** *P* < 0.01,*** *P* < 0.001, vs control cells. **Fig. S2** Complementary results: Impact at 30 days of the removal of Tat and/or Nef after 20 days of treatment. MSCs were treated with the HIV proteins Tat and/or Nef, either up to 30 days or up to 20 days and then the proteins were removed up to day 30. Mean PDL values (± SEM), were determined at the indicated days post-HIV-proteins treatment (A). The % inhibition of population growth was calculated for each HIV protein at day-30 compared to control cells (B). SA- β-galactosidase activity was evaluated and expressed as the total % of positive cells at pH6 (C). Lysosomal accumulation was assessed by LysoTracker fluorescence probe and expressed as % of control cells (D). ROS production was assessed by the oxidation of CM-H_2_DCFDA (E) and expressed as % of control cells. Mitochondrial mass was evaluated by MitoTracker Red-Probe (F), and mitochondrial membrane potential by JC-1 fluorescent dye (G). Results are mean ± SEM. All experiments were performed in duplicate or triplicate in MSCs isolated from 3 different bone marrow donors. * *P* < 0.05, ** *P* < 0.01,*** *P* < 0.001, vs control cells. **Fig. S3** Complementary results: Nef impedes the autophagic efflux in MSCs: prevention by rapamycin. Human BECN1, GABARAP and ULK mRNA levels were measured using real time RT PCR on day-20 post HIV proteins treatment. The relative mRNA expression levels were normalized to PPIA (A). Whole-cell lysates were extracted from MSCs at day-10 and day-20 of treatment with the HIV proteins Tat, Nef and Tat/Nef, and analysed by immunoblotting. Representative immunoblots of Akt and Phospho Akt are shown (B). Rapamycin (Rapa) was added from day 15 to day 20. Mean cell number, and the per cent inhibition of population growth were calculated for each condition (C). Mitochondrial mass was evaluated by MitoTracker Red- Probe (D), and mitochondrial membrane potential by JC-1 fluorescent dye (E). Results are mean ± SEM. All experiments were performed in duplicate or triplicate. * *P* < 0.05, ** *P* < 0.01,*** *P* < 0.001, *vs* control or *vs* Tat- and/or Nef-treated cells without rapamycin. NS, non significant.
