# Supplementary figures and images for: The HIV proteins Tat and Nef promote human bone marrow mesenchymal stem cell senescence and alter osteoblastic differentiation
Source: Aging Cell. 2015 Apr 7;14(4):534–46. doi: 10.1111/acel.12308 (PMC4531068; doi:10.1111/acel.12308)

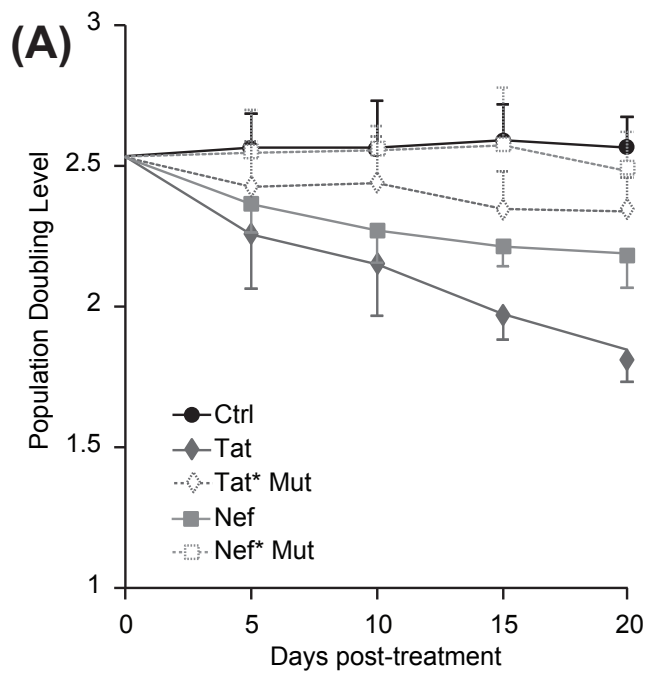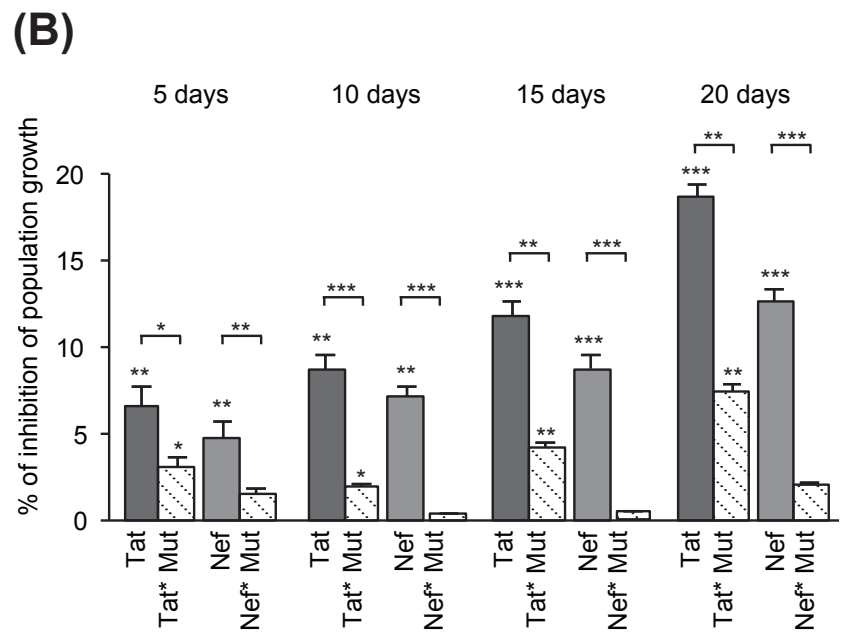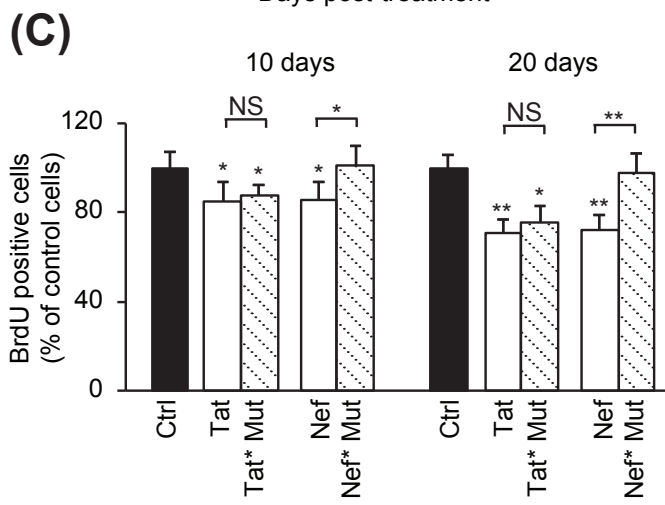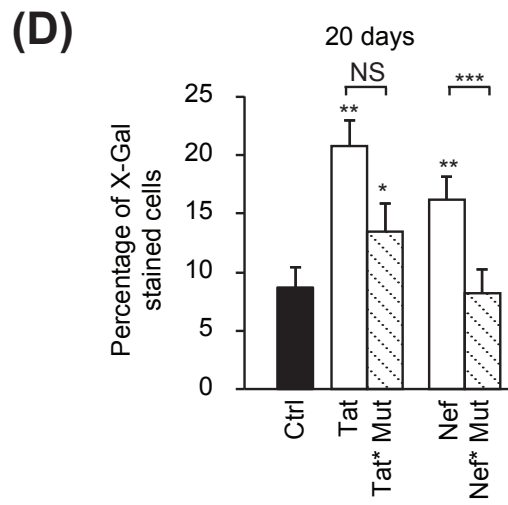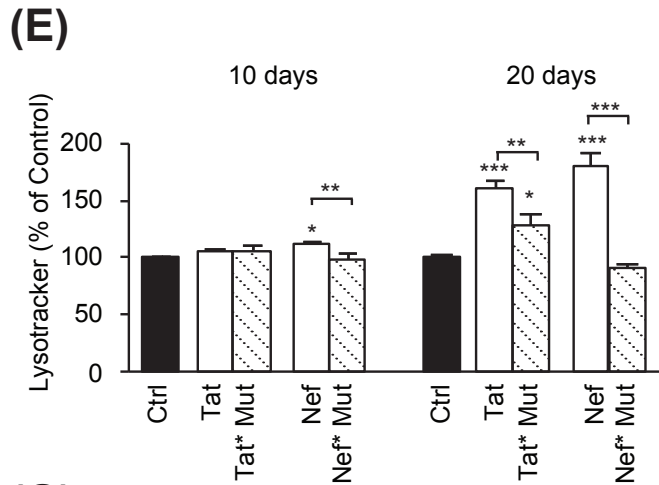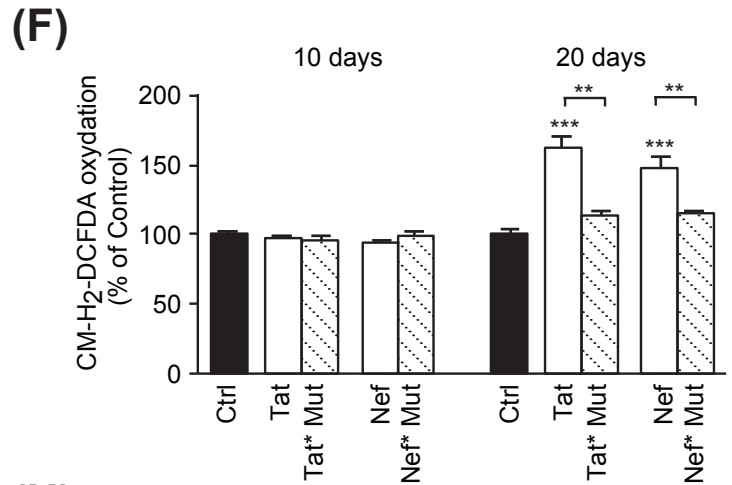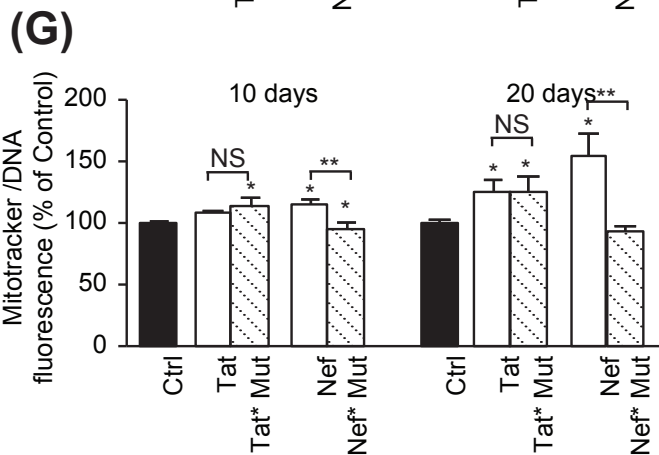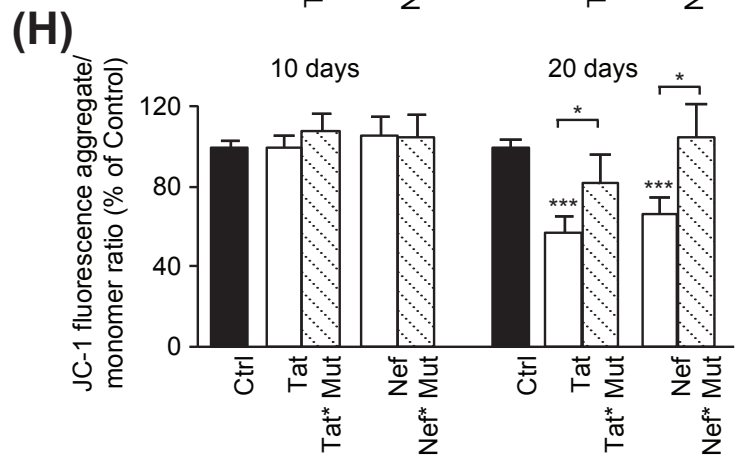

**(A)**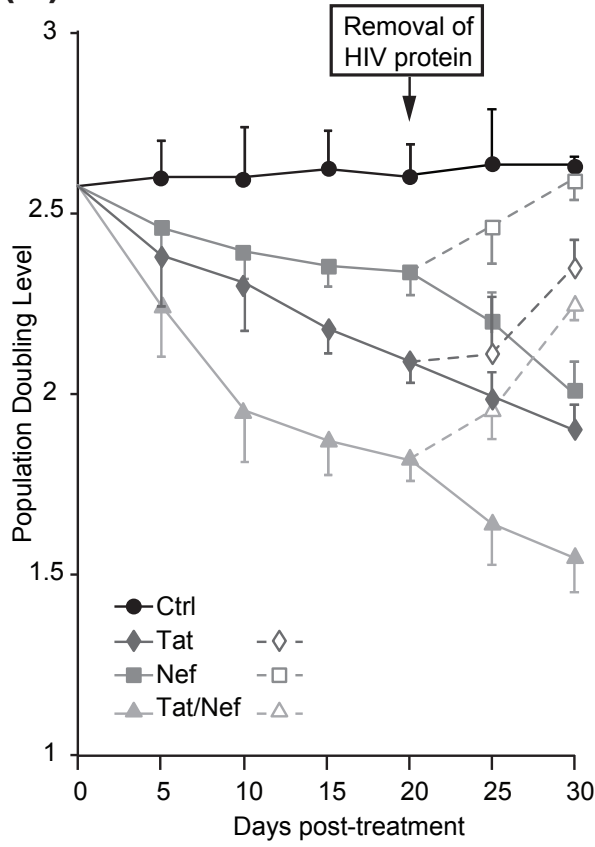**(B)**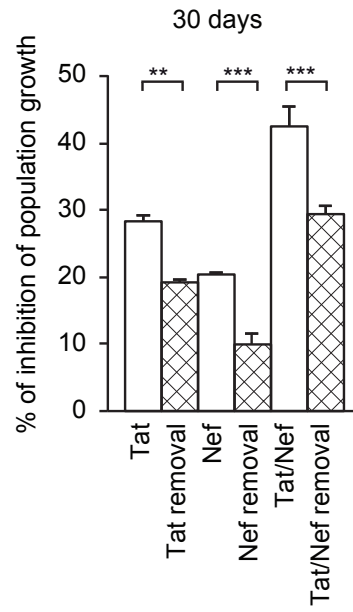**(C)**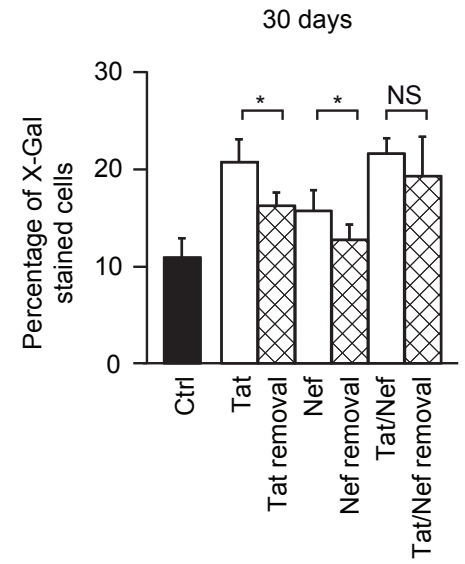**(D)**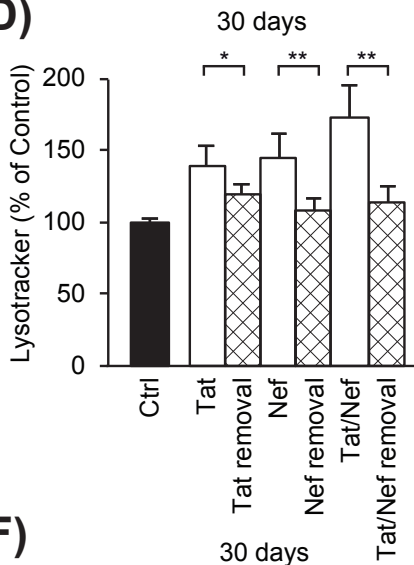**(E)**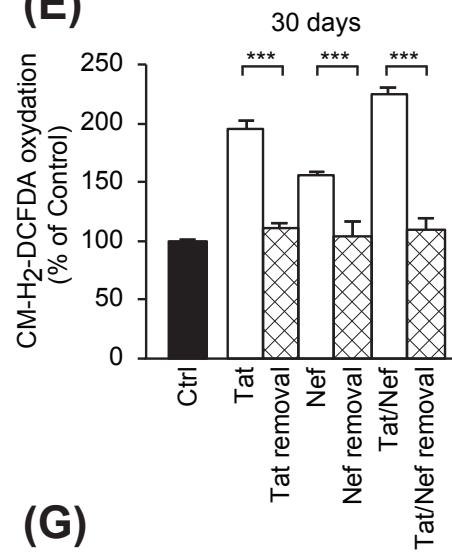**(F)**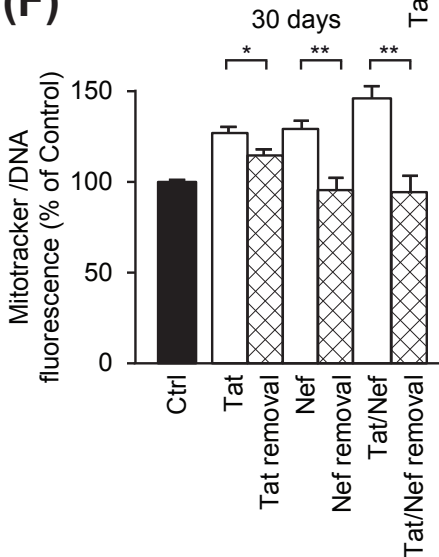**(G)**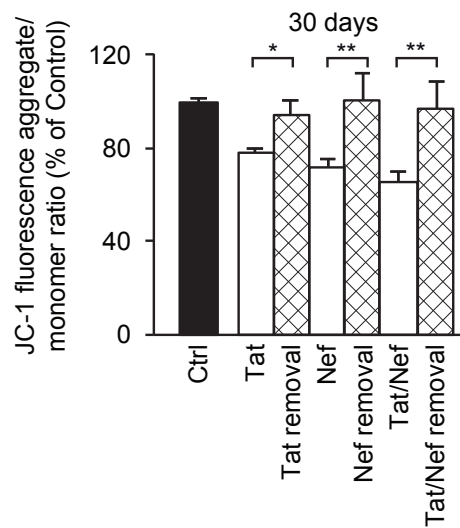

**(A)**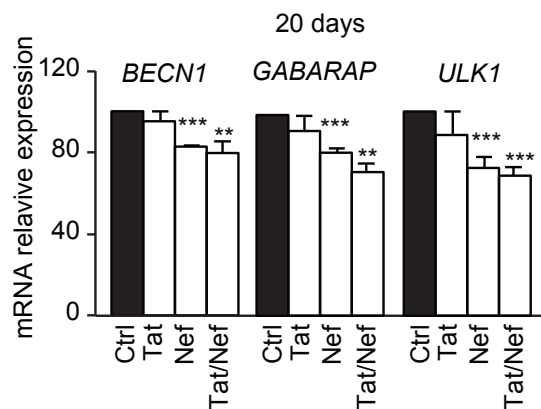**(B)**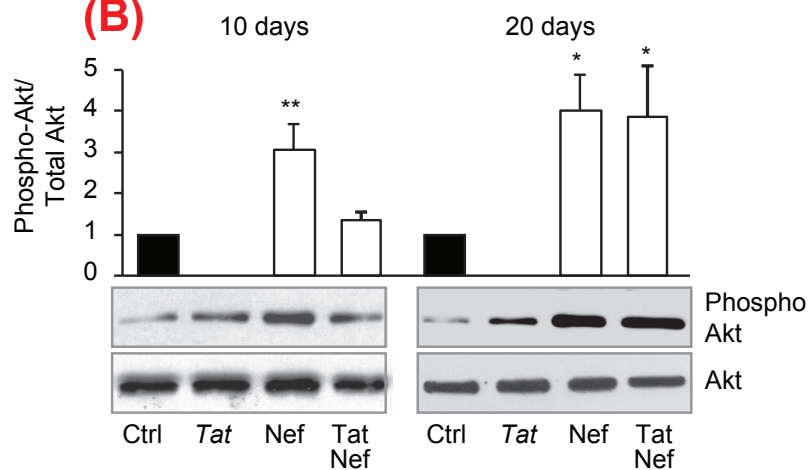**(C)**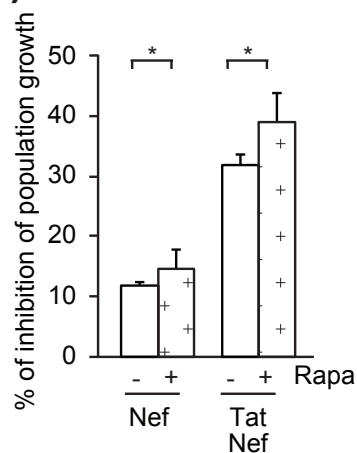**(D)**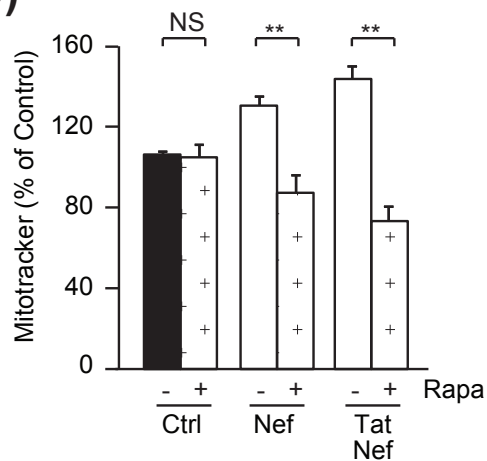**(E)**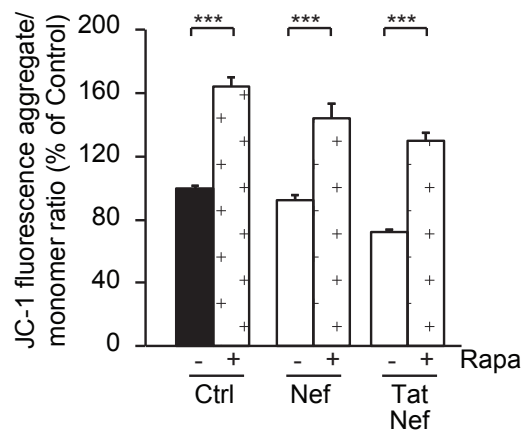

Supplement: Supplementary file 1 [file acel0014-0534-sd1.pdf]
